# Clonidine: An Old Friend Newly Rediscovered

**DOI:** 10.5812/kowsar.22287523.1802

**Published:** 2011-07-01

**Authors:** Ali Dabbagh

**Affiliations:** 1 Anesthesiology Research Center, Shahid Beheshti University of Medical Sciences, Tehran, IR Iran

**Keywords:** Clonidine, Anesthesia, Analgesics

A number of decades have passed since the beginning of clinical use of clonidine. Nowadays, there is growing role defined for clonidine, an alpha-2 adrenergic agonist, used originally as an antihypertensive drug and then, as an oral, intravenous or now, an intrathecal agent. This drug would surpass the blood-brain barrier to target the central alpha 2 adrenoceptors to affect the CNS by decreasing the brain sympathetic tone; this would result in decreased systolic and diastolic blood pressure and heart rate (1-3). However, central alpha 2 adrenoceptor agonistic effects is not the only route for clonidine, because the drug has intrathecal effects when administered as an adjuvant during spinal or epidural anesthesia (combined with local anesthetics). Besides, its peripheral effects have been described fully (3). There are a number of studies well demonstrating that oral clonidine as a pretreatment to anesthesia, can increase the quality of perioperative sedation and analgesia while having just a few side effects (3-5). These beneficial effects of clonidine have been demonstrated not only in adult patients but also, in children (4) and both in patients undergoing general anesthesia (1), neuraxial block (5-7) and nerve blocks (8). Even, there are studies that have claimed the role of neuraxial clonidine in decreasing the chronic intractable pain after thoracotomy (9). But the newly performed studies have claimed that the peripheral and neuraxial effects of clonidine may have some different mechanism of action rather than the previously demonstrated role of clonidine as an alpha 2 adrenoceptor agonist. Though the exact mechanism is not clear, some hypothetical mechanisms have been proposed and a number of them like the role of clonidine in inhibition of tetrodotoxin-sensitive sodium channels have been demonstrated (10). Much more similar studies like the latter one are in process. Maybe one of the most prominent features of clonidine use in anesthesia and analgesia is the newly discovered drug mechanisms and more than that, the newly found clinical methods used for clonidine as an adjuvant for systemic and regional anesthetic drugs. Though dexmedetomidine, a synthetic analog of clonidine is widely available with a number of superiorities, clonidine, especially the oral form of the drug, remains in the mainstay as an anesthetic in clinic and in research. Comparing the cost effectiveness issues, clonidine is possibly much more advantageous. However, the newly discovered mechanisms of drug effect (both for acute pain suppression and chronic pain alleviation and also, as an anesthetic) remain as active fields of research.
